# Imaging of Deep Venous Pathology

**DOI:** 10.1007/s00270-024-03785-y

**Published:** 2024-07-01

**Authors:** Carsten W. K. P. Arnoldussen

**Affiliations:** Interventional and Cardiovascular Radiologist, Department of Diagnostic and Interventional Radiology and Nuclear Medicine, VieCuri Medical Centre, Tegelseweg 210, 5912 BL Venlo, Limburg The Netherlands

**Keywords:** Deep vein thrombosis, DVT, Chronic deep vein obstruction, Post-thrombotic syndrome, PTS, May-Thurner syndrome, NIVL, Nuttcracker syndrome, Thrombolysis, Thrombectomy, Pharmacomechanical thrombolysis, Recanalization, Venous stenting, Magnetic resonance venography, Computed tomography venography, Duplex ultrasonography, DUS, Intravascular ultrasound, IVUS, Conventional venography

## Abstract

Imaging plays an important role in the identification and assessment of clinically suspected venous pathology. The purpose of this article is to review the spectrum of image-based diagnostic tools used in the investigation of suspected deep vein disease, both obstructive (deep vein thrombosis and post-thrombotic vein changes) as well as insufficiency (e.g., compression syndromes and pelvic venous insufficiency). Additionally, specific imaging modalities are used for the treatment and during clinical follow-up. The use of duplex ultrasound, magnetic resonance venography, computed tomography venography and intravascular ultrasound as well as conventional venography will be discussed in this pictorial review.

## Diagnosis and Imaging of Deep Vein Thrombosis and the Post-thrombotic Syndrome

It is important to understand the reasons why imaging has become such a crucial part of the assessment of patients with an acute deep vein thrombosis (DVT) and the potential long-term sequelae referred to as the post-thrombotic syndrome (PTS). The symptoms and signs of venous thrombosis are caused by obstruction to venous outflow, vascular inflammation, or pulmonary embolization. Physicians cannot rely solely on clinical signs and symptoms to establish diagnosis and extent of deep vein thrombosis, and must therefore depend on imaging studies to guide treatment [1]. In 60–80% of patients referred for clinically suspected venous thrombosis, however, diagnosis will not be confirmed by objective testing, stressing the clinical complexity of diagnosing deep vein thrombosis.

Duplex ultrasound (DUS) is the noninvasive diagnostic modality most often used for diagnosis of deep vein thrombosis, with a reported sensitivity and specificity of approximately 97% [2]. Other imaging modalities that are currently used to diagnose deep vein thrombosis include computed tomography (CT), and magnetic resonance imaging (MRI) [3, 4].

It is essential to accurately diagnose deep vein thrombosis as soon as possible to start the treatment early. Early treatment of deep vein thrombosis with anticoagulants has been demonstrated to reduce the incidence of pulmonary embolism and associated mortality [5]. Furthermore, early treatment prevents extension of deep vein thrombosis from distal veins to more proximal veins and relieves acute symptoms in the leg [6, 7]. Rapid achievement of therapeutic anticoagulation and adequate treatment duration prevent early recurrence of deep vein thrombosis and may also decrease the incidence of post-thrombotic syndrome [7].

## Duplex Ultrasound

DUS is well established as the imaging modality of choice for the assessment of deep vein thrombosis [11]. Examinations are non-invasive, rapidly obtained, and can be performed serially.

In symptomatic patients, as mentioned above, venous compression US has a reported sensitivity and specificity of approximately 97%. However, DUS is less sensitive for calf vein thrombosis, pelvic (ilio-caval) thrombosis and regarding asymptomatic DVT occurring after surgery [12, 13]. Sensitivity for calf vein thrombosis increases using duplex in addition to compression [14]. Also, patients with symptoms of recurrent DVT are a difficult diagnostic challenge [1]. Only about 20–30% of these individuals actually do have recurrent venous thromboembolism (VTE); the remainder has symptoms arising from chronic venous insufficiency or other causes (most common reported examples: muscle rupture (18.5%), chronic venous insufficiency (CVI) (14.6%), erysipelas/cellulitis (12.6%) and superficial venous thrombosis (SVT) (10.9%) [15]. After an acute episode, DUS shows abnormalities indistinguishable from the original findings of deep vein thrombosis for a duration of 6 months in up to 50% of patients [16]. Hence, there are a significant number of patients and clinical circumstances in which the diagnosis of (acute) deep vein thrombosis is difficult to establish with DUS. Acute occlusion of the pelvic veins and the inferior vena cava, often due to thrombus extension from the femoropopliteal system, represents a major risk for pulmonary embolism (PE) and forms another diagnostic challenge [6]. Color flow Doppler imaging is often limited for the diagnosis of iliocaval thrombosis owing to obesity, bowel gas and deep location of pelvic veins. Severe edema, skin breakdown, or the presence of a cast may limit the accuracy of DUS.

**Fig. 1:**
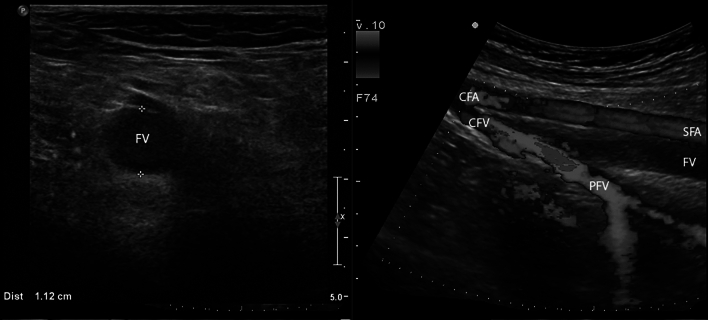
Axial DUS image (left) of the femoral vein (FV). The FV is dilated and completely obstructed with thrombus (hypo-echogenic material). Saggital color duplex image (right) of proximal FV thrombosis. Image recorded while manually compressing the (lower) leg to augment flow and show the (adequate) inflow from the profunda femoral vein (PFV) into the common femoral vein (CFV)

**Fig. 2:**
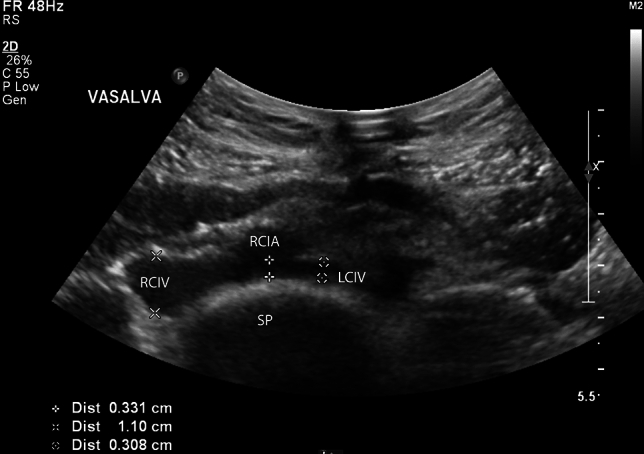
DUS image showing the compressed and stenotic left common iliac vein (LCIV, 0.3 cm) compressed between the right common iliac artery (RCIA) and the lumbar spine (SP). Image recorded while performing Vasalva maneuver illustrating the marked difference in diameter in comparison to the right common iliac vein (RCIV, 1.1 cm)

**Fig. 3:**
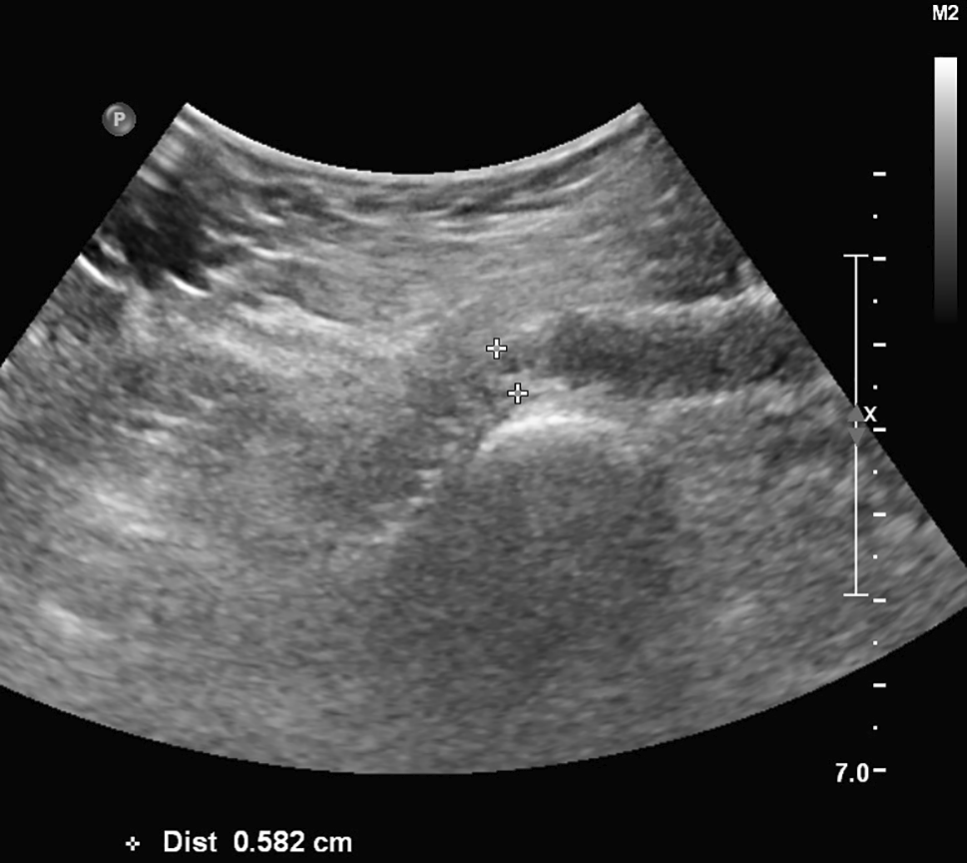
DUS image showing a stent compression at the level of the inguinal ligament (diameter reduction 50%) at 1 year FU after recanalization and stenting of a chronically occluded common iliac, external iliac and proximal common femoral vein

While studies on the comparison of imaging techniques after intervention are virtually non-existent, DUS offers a good starting point to evaluate the patency of the venous system after interventions. Both in- and outflow through (central) venous stents as well as lumen patency of the deep vein system of the leg can routinely (and serially) be performed with DUS. (Figs. 1, 2, 3).

## Computed Tomography/Computed Tomography Venography

CT can be used to accurately diagnose acute pelvic vein or inferior vena cava occlusion and are additionally also capable of diagnosing pulmonary emboli, offering a single diagnostic examination to assess both [17–19]. Most commonly used is multidetector computed tomography angiography (CTA), combined with late venous-phase imaging (indirect CTV), to accurately diagnose a pelvic vein or inferior vena cava occlusion, which can be the source of significant pulmonary emboli. On CTA/CTV thrombi appear as intravascular hypodense masses, sometimes encircled by a hyperdense rim of contrast medium. The thrombosed veins are generally dilated compared to their normal dimensions. In some cases, CT is capable of distinguishing perivascular stranding as a sign of an antithrombotic/inflammatory response. The reported specificity and sensitivity compared to DUS are approximately 93–100% and 97%, respectively [17, 18]. Indirect CTV, for which contrast is injected through a cubital vein and scanning is performed at 70–90 s post-contrast injection, in addition to CT pulmonary angiography is a relatively accurate method for evaluation of femoropopliteal venous thrombosis in one examination without the need for a second contrast bolus [19]. But introducing CTV as an alternative to DUS is not ideal since it introduces risks associated with radiation and iodine contrast material. The use of MRI is more appealing in this regard, as MRI is also noninvasive, but does not require radiation or iodinated contrast material and can also be used in specific risk groups such as pregnant women [19].

**Fig. 4:**
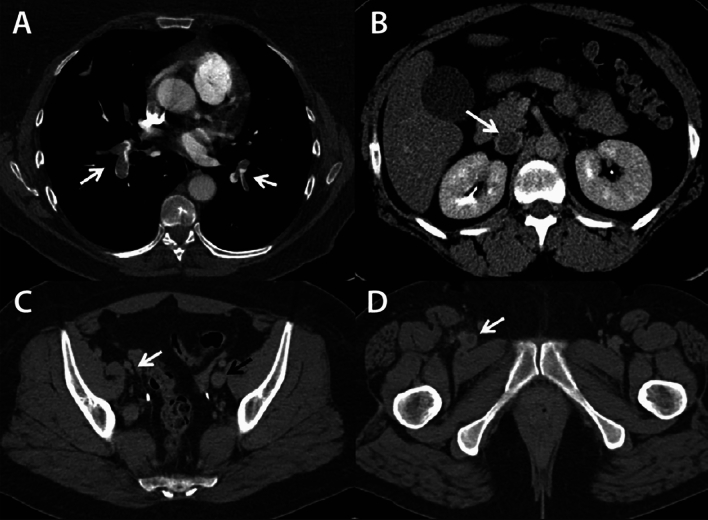
CTPA showing massive bilateral PE (**a**). Indirect CTV demonstrates acute IVC thrombosis (**b**), right external iliac vein scarring due to previous DVT (**c**), and right common femoral vein thrombosis (**d**).—*courtesy of dr A.M. Alduk, MD, PhD, Department of Diagnostic and Interventional Radiology, University Hospital Center Zagreb, Croatia & professor G. O’Sullivan, MD, Department of Interventional Radiology, University hospital Galway, Ireland*

**Fig. 5:**
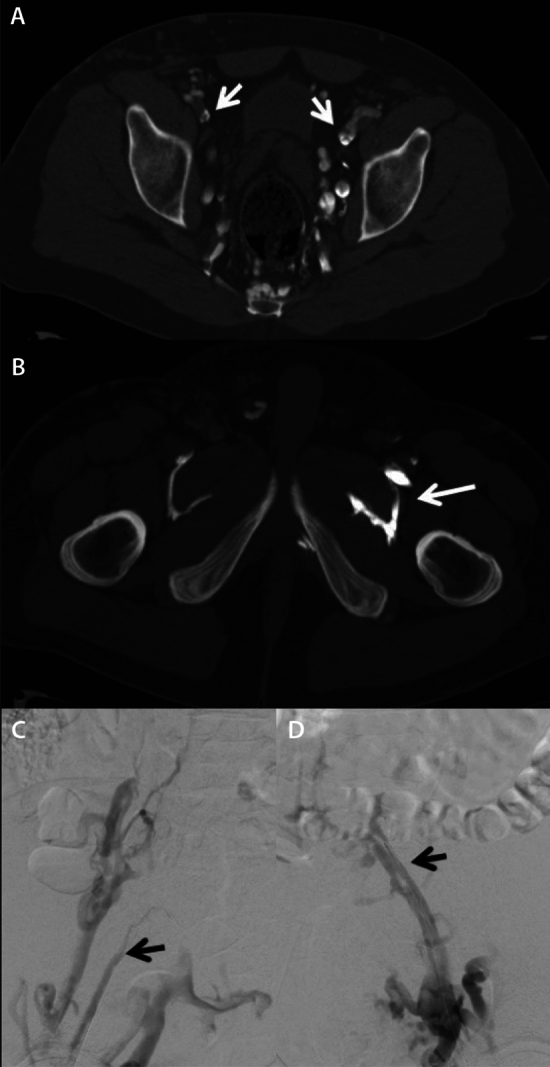
direct CTV shows chronic bilateral iliac vein occlusion (**a**) with typical ‘obturator hook’ sign; this sharp angled vein configuration with obturator vein enlargement is a marker for hemodynamically significant collateralization between external iliac vein or common iliac vein (CIV) and internal iliac vein, representing therefore an indirect sign of chronic iliac vein outflow obstruction. (**b**). Right (**c**) and left (**d**) iliac venograms show good correlation with direct CTV.—*courtesy of dr A.M. Alduk, MD, PhD, Department of Diagnostic and Interventional Radiology, University Hospital Center Zagreb, Croatia & professor G. O’Sullivan, MD, Department of Interventional Radiology, University hospital Galway, Ireland*

**Fig. 6:**
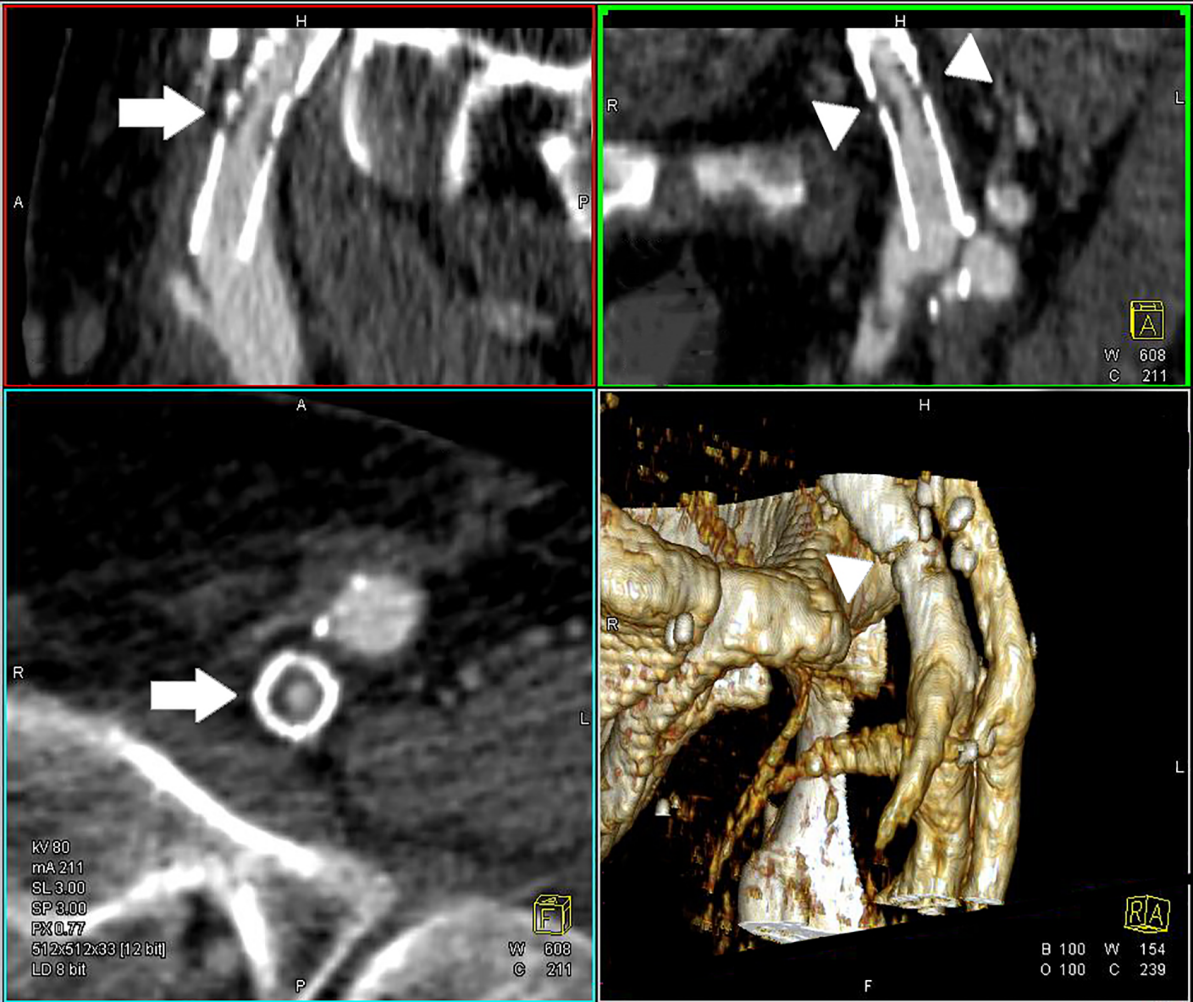
CTV image showing in-stent restenosis at the level of the inguinal ligament (arrows) with a stent fracture and dislocated stent struts (arrowheads). The 3D volume rendering also shows the stent fracture (bottom right picture, arrowhead)

For chronic obstruction, CTV, in particular direct CTV, can be utilized in addition to DUS. The reason direct CTV is to be preferred over indirect CTV is the lack of intraluminal detail with indirect CTV, which are vital components of the post-thrombotic disease, both for detection of disease and pre-interventional planning. There are different approaches to performing direct CTV. Either with or without a tourniquet. Without a tourniquet, a thigh-high compression stocking is placed on the affected limb, keeping the foot out. Then a 20-gage venous cannula is inserted into a vein in the dorsum of the foot (or ankle). Up to 100 mL of iodinated contrast is injected at 3 mL/s with a 30-mL saline chaser. The scan volume should cover mid-calf to the diaphragm. Experienced centers have shown added value for direct CTV in chronic venous obstruction [21]. CTV should be limited in young patients, since repeated imaging is likely to be required. The same applies to pregnant patients and in patients with total hip replacements or lumbar spine surgery with metallic implants in which metal hardening artifacts are an issue. (Figs. 4, 5, 6).

It is common practice to follow-up patients after interventions with DUS. MRV is generally inaccurate for assessment of in-stent thrombosis or stenosis. If stent-related complications are suspected or detected, CTV in particular is capable of a more precise analysis of the (stent-related) problem. CTV has a high spatial resolution which contributes to highly detailed imaging as depicted in Fig. 6 allowing for assessment of the stent position and integrity, the lumen and (the degree of) (re)stenosis.

## Magnetic Resonance Imaging

MRI is superior in soft tissue contrast and offers several different scanning techniques. Two-dimensional time-of-flight venography (TOF-MRI) has the potential to perform a magnetic resonance venography (MRV) without contrast. Thrombotic material is depicted as a hypointense filling defect or, alternatively MRI can detect a thrombus directly (MR direct thrombus imaging or MR-DTI). This technique can be applied to various T1 sequences (for example 3D T1 TFE, SPAIR, FFE or THRIVE), relying on a shortened relaxation time to detect methemoglobin, enabling direct visualization of pulmonary emboli and leg vein thrombi without the need for intravenous contrast. This signal is present in the acute phase and decays over time (disappears over a period of 3–6 months). Compared to DUS and contrast venography, this method has been reported to have a sensitivity of 98% and a specificity of 96% [22]. MRI accuracy can be further improved by adding gadolinium-based contrast agents (MR angiography in the venous or steady-state phase, referred to as MRV). In a study designed to evaluate the diagnostic value of MRV and DUS in the assessment of deep vein thrombosis compared with contrast-enhanced venography, MRV was 100% sensitive and 100% specific in diagnosing deep vein thrombosis above the knee [23]. With MRV it is also possible to differentiate between acute occlusion and chronic thrombosis, and it has also shown to be more accurate than DUS in detecting extension of deep venous thrombosis [4, 24]. MRV also correctly depicts venous anatomy and patency of the central veins. Therefore, MRV should be considered the modality of choice for the evaluation of venous occlusion of the large systemic veins (e.g., inferior vena cava, pelvic veins, superior vena cava, subclavian veins and/or other deep chest/abdominal veins).

**Fig. 7:**
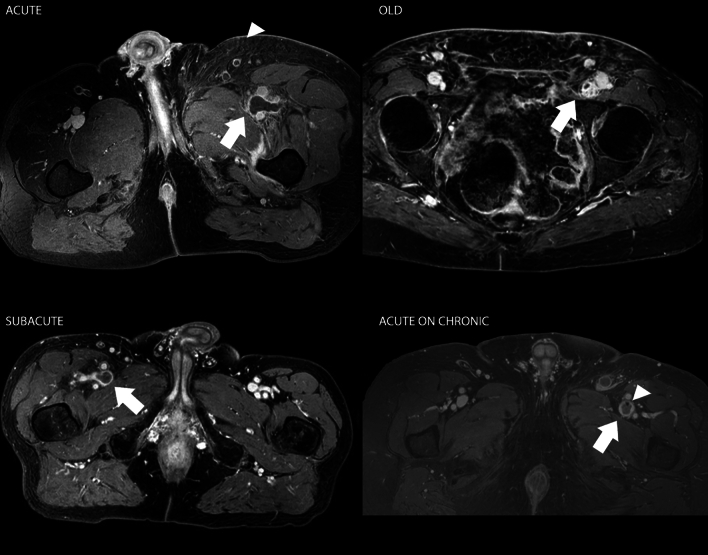
CE MRV depicting deep vein thrombosis in various distinguishable stages of thrombus evolution in time. Acute: hypointense signal in the thrombus in the dilated common femoral vein with a thin enhancing rim of contrast in the vein wall. Note the soft tissue edema in the subcutaneous layers (arrowhead). Subacute: more heterogeneous signal intensities in the thrombus in the common femoral vein and a more thickened vein wall with some perivascular hyperintense signal. Old: hypointense signal in what appears to be a remnant of thrombus with prominent enhancement in and around the vein wall. Acute on chronic: hypointense signal in the thrombus in the common femoral vein with marked enhancement in what appears to be a thickened vein wall. Note the black dot (arrowhead) which is the typical appearance of post-thrombotic sequalae and/or fibrotic scar tissue from a previous thrombotic event in the past

**Fig. 8:**
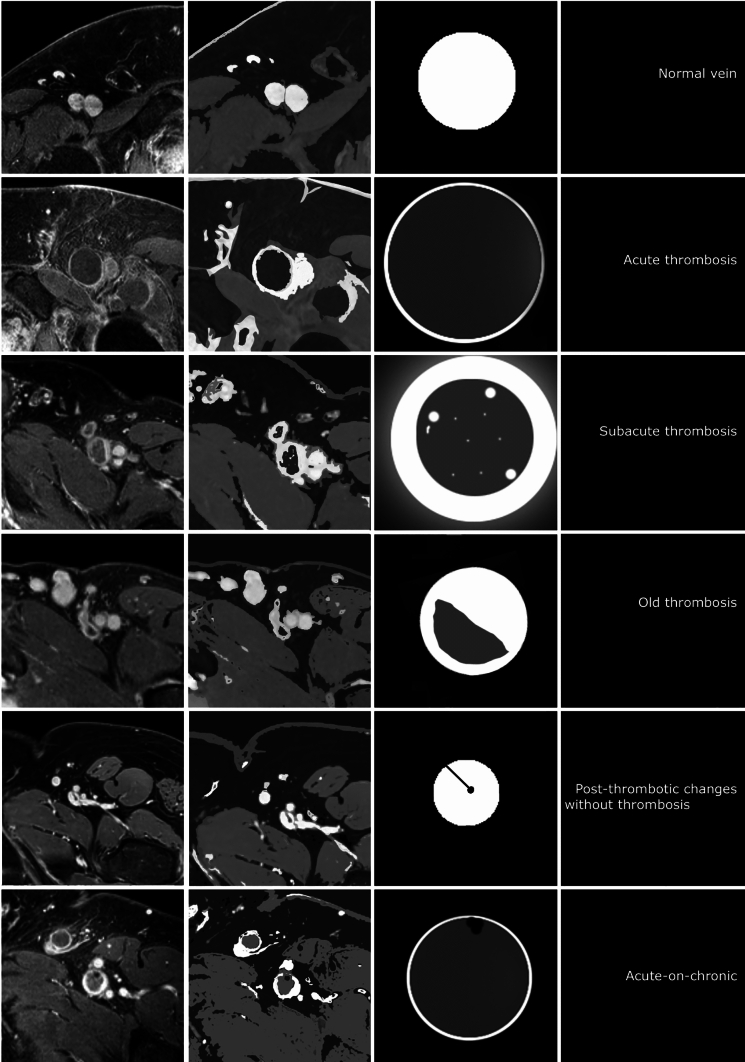
More detailed interpretation of thrombus characteristics with MRV: Normal vein: homogeneously opacified hyperintense vein lumen. No luminal defect or perivascular) wall changes. Acute thrombosed vein: dilated homogeneously hypointense vein lumen with small enhancing rim of contrast depicting the vein wall. No (perivascular) wall changes (no halo sign). Subacute thrombosed vein: Still dilated low intensity vein lumen with thick enhancing rim of contrast, part vein wall thickening and part perivascular edema (halo sign). There are some small hyperintense areas within the thrombus as sign of recanalization. ‘Old’ thrombosed vein: the vein lumen is reduced to a more ‘normal’ vein size with an opacified part (open lumen/vein wall) and a low intensity part that is still filled with remnant thrombus-like tissue. Post-thrombotic vein: the vein lumen is smaller than the normal vein and homogeneously opacified except for 1 or more sharply demarcated very low intensity black dots and/or lines adhering to the vein wall. This represents (fibrotic) scar tissue (post-thrombotic venous scarring). Acute-on-chronic thrombosed vein: as in an acute deep vein thrombosis there is a dilated lumen with mostly hypointense material but additionally there are signs of a previous thrombotic event that has left scar tissue markings (very hypointense dots and lines)

In a recent publication, it was shown that thrombus aging based on MRV imaging enables preprocedural selection of patients with iliofemoral DVT most likely to undergo successful catheter-directed thrombolysis (CDT), as well as those most likely to be resistant to thrombolytic therapy [26]. This helps to avoid unnecessary risk associated with unsuccessful catheter-directed thrombolysis and extensive treatment duration. This shows the potential for MRV to assist in patient selection for more invasive treatment options in addition to anticoagulant therapies (Figs. 7, 8).

However, as it stands, MRV has not been established as superior to DUS for the general diagnosis of deep vein thrombosis in the arms or legs by peer reviewed medical literature. MRV has not been shown to be superior to DUS for lower limb deep vein thrombosis, except for imaging the deep femoral and hypogastric vessels [23, 25, 26]. Currently, information about these vessels is not routinely used in therapeutic decisions, except in patients with pulmonary emboli where the source of the emboli could not be identified using DUS [6].

Considering the risks associated with and limitations of CTV, MRV is the most suitable alternative to DUS in inconclusive cases and can additionally aid interventionalists in their preprocedural work up and patient selection. However, following stent placement, MRV is inaccurate for assessment of in-stent thrombosis or stenosis and either DUS or CTV is preferred.

## Venography and Intravascular Ultrasound

In most practices conventional venography and IVUS are reserved for the interventional imaging rather than diagnostic imaging stage. Venography, while formally still the gold standard for most aspects of the diagnostic evaluation, is primarily used to guide the intervention and perform controls. The main disadvantages of venography with regard to its value as a diagnostic tool are its invasive nature, the need for ionizing radiation and iodine contrast material. For diagnostic purposes, CTV has the same disadvantages but offers a 3D anatomic overview. Additionally, with all the 3D tools available, in particular following the introduction of intravascular ultrasound (IVUS), the limitations of 2D venography, even in multiple projects are becoming increasingly evident [28]. One of the major diagnostic challenges that remain is the identification of patients which based on (a combination of) clinical presentation and images findings are most likely to benefit from specific treatment. A study by Gagne et al. published in 2018 [29] has shown the potential for IVUS to identify iliac vein stenosis and measure the degree of stenosis, in combination with a clinical, etiologic, anatomic and pathophysiologic (CEAP) classification of 4–6 to have a predictive value for symptom improvement after stenting. Additionally, it is common sense to evaluate stenotic lesions in 3D rather than 2D to assess their ‘true’ degree of stenosis. However, MRV and CTV should be proven to be as capable as IVUS for this 3D assessment, unfortunately currently studies investigating this in more detail have not yet been published. The current evidence available on CTV suggests at least there is value in screening patients prior to more invasive investigations [30].

Summarizing the approach to evaluating venous stenotic/obstructive disease, DUS is the most convenient initial test, but in a scenario with a high clinical suspicion of a stenosis, inconclusive or negative DUS findings should be followed with either MRV or CTV to better visualize the entire venous system, in particular the more proximal vessels. Additionally, these imaging techniques show the entire anatomy, which may highlight other causes of extrinsic compression. Invasive imaging should be reserved for patients with negative findings on non-invasive imaging with a persistent high clinical suspicion for venous outflow obstruction (stenosis/occlusion).

**Fig. 9:**
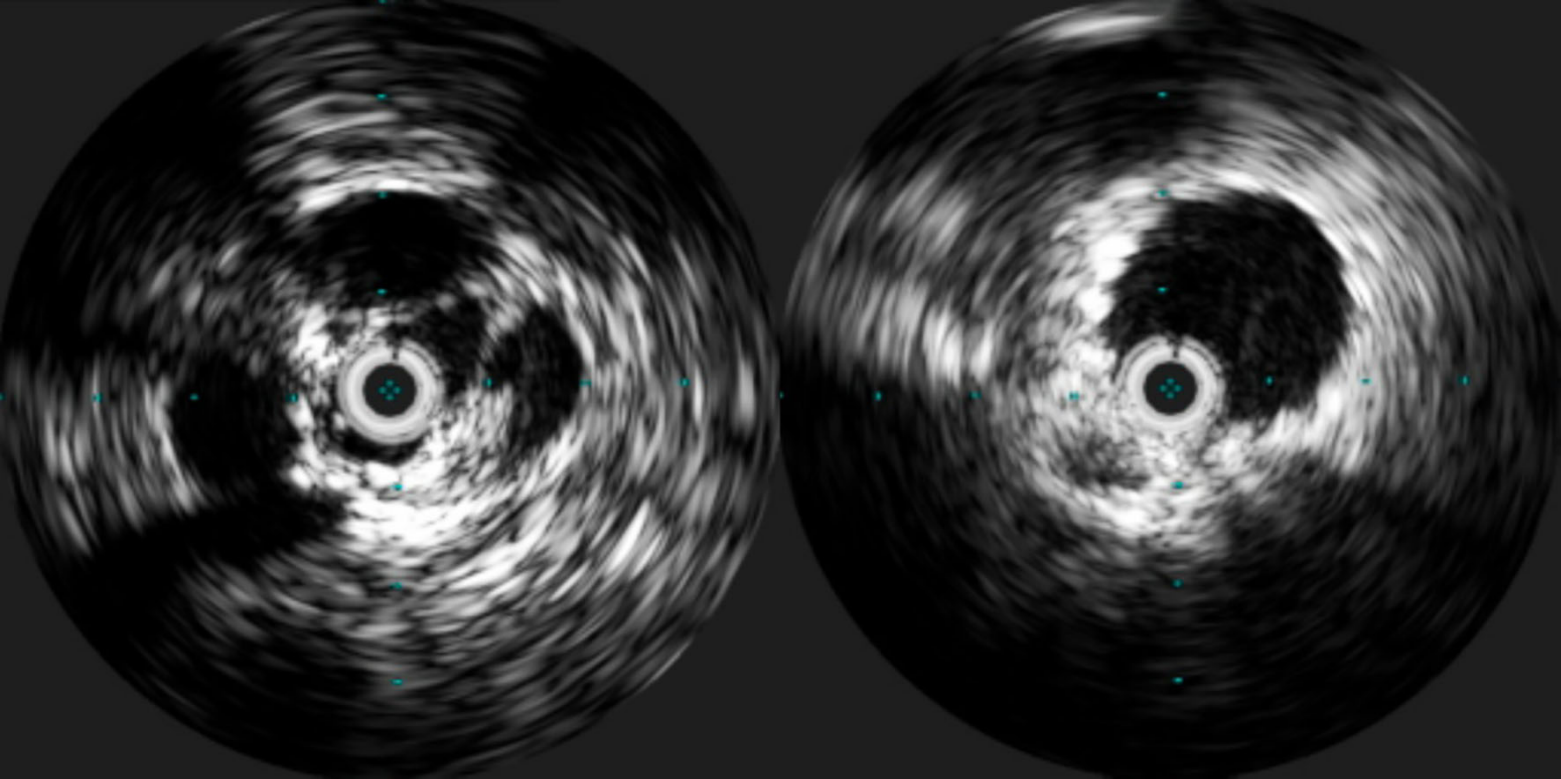
IVUS images obtain prior to (left) and after stent placement (right) of the common iliac vein. Notice the luminal changes on the left and the open vein on the right with the stent struts in apposition to the vein wall

On the other hand, there are advantages to both venography and IVUS during the procedure. Both are real time and allow for patient position manipulation to mimic dynamics while still in a supine position. Where conventional venography, being limited to 2-dimensional visualization falls short, with IVUS it is possible to evaluate the veins in 3D allowing for evaluation of residual outflow obstruction and very precise stent placement. This can be used to prevent the coverage of the contralateral iliac vein outflow tract while stenting the common iliac vein, as well as when choosing the right landing zone distally above the confluence of the femoral and deep femoral vein [31] (Fig. 9).

Future developments might lead to an interventional suite requiring less radiation and iodine contrast material, limiting fluoroscopy time with the aid of not only IVUS but also improved multimodality fusion techniques, using the imaging studies from the diagnostic evaluation in the interventional setting.

## Diagnosis and Imaging of Pelvic Venous Disorders

Pelvic venous disorders (PeVD) cover a spectrum of signs and symptoms from the abdomen, pelvis and legs [30, 33]. From an imaging perspective, PeVD are made up from 2 different conditions. The first is venous reflux, causing a hemodynamic shift in the abdomen/pelvis. The second is due to obstruction in the deep veins impeding outflow. The result of either or both these conditions is dilation and dysfunction of the pelvic veins characterized by slow flow and reflux [34].

Symptoms from PeVD are variable and in fact, superficial varicose veins may occur without any pelvic symptoms and thus may be the only findings in PeVD. The pelvic circulation is highly complex and interrelated, which contributed to misdiagnosis and very variable outcomes in the clinical research published to date [34]. The concept of PeVD, proposed by Meissner et al., centers around the attempts to address the complexity of the problem [33]. A similar degree of venous insufficiency may produce different symptoms, as well as identical symptoms may have different underlying pathophysiology in different patients [33]. It is important to acknowledge that superficial varicose veins may occur without any pelvic pain and be the only symptom of PVI [36].

**Fig. 10:**
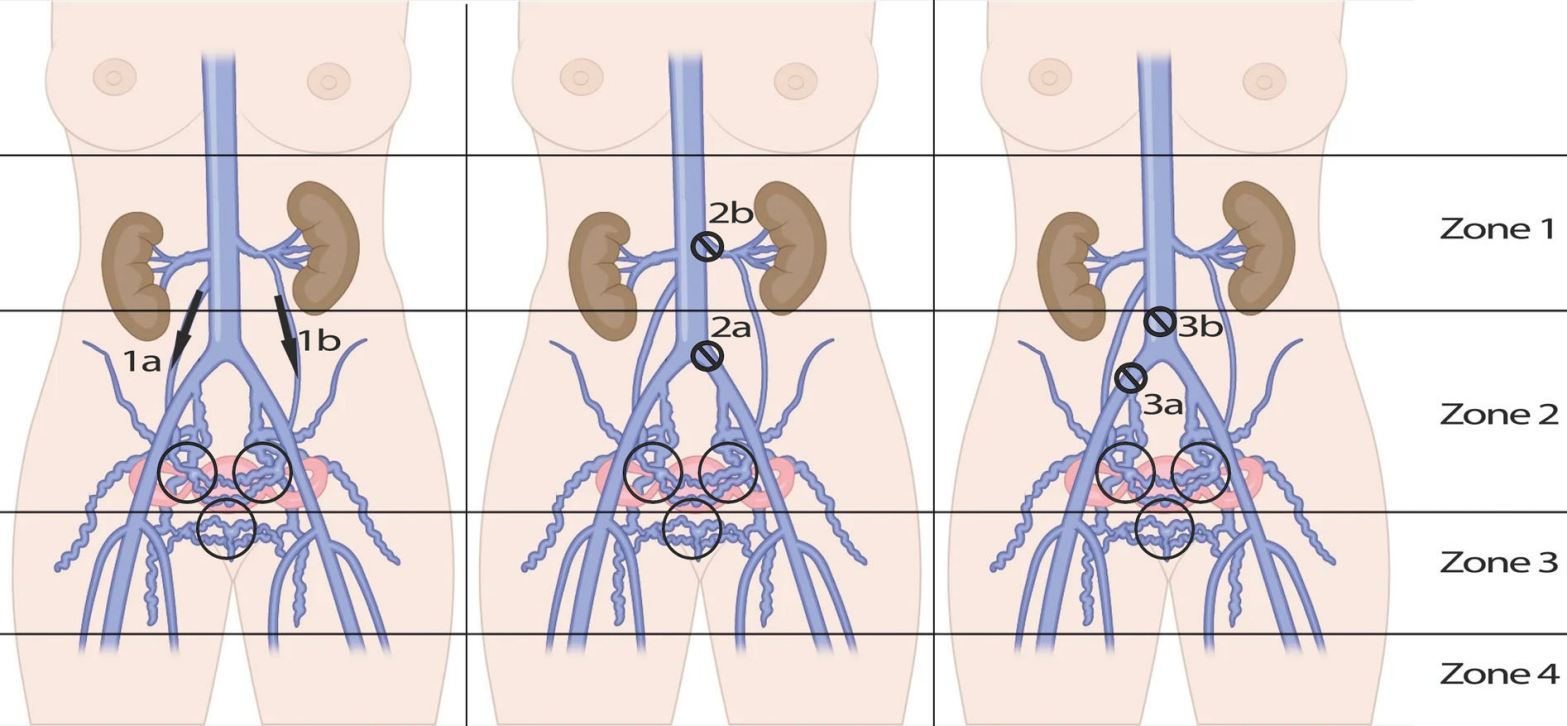
Schematic classification of pelvic venous disorders (PeVD). (**a**) insufficiency. Type Ia: unilateral venous insufficiency. Type Ib: bilateral venous insufficiency. (**b**) compression. Type IIa: May-Thurner syndrome. Type IIb: nutcracker phenomenon. (Type IIc: both IIa and IIb). (**c**) obstruction. Type IIIa: common iliac vein obstruction. Type IIIb: inferior vena cava obstruction. Type IId (extrinsic compression), IIIc (portal hypertension), IV (arteriovenous malformation or fistula). and V (nutcracker Syndrome) are not displayed

Hence PeVD should be regarded as the basket of possible vein changes resulting in chronic (pelvic) complaints, caused by either pelvic venous insufficiency (for example the pelvic congestion syndrome (PCS)) and/or Nutcracker Syndrome (NTS)) or variations of the May-Thurner Syndrome (MTS) and/or chronic obstruction. The classification proposed by Meissner et al. categorizes these disease entities or patterns based on symptoms (S), varicosities (V) and the underlying pathophysiology (P), referred to as the SVP-classification. [32] The goal of the classification is, in addition to helping clinicians to categorize the (type of) disease, to be able to compare treatment strategies for specific disease entities. The concept facilitates a comparison of similar patterns of symptoms, underlying varicosities and pathophysiologic abnormalities in all aspects of the disease by standardizing the reporting. Examples of the same areas being affected by different pathophysiology are shown in (Fig. 10).

## Role of Imaging

Imaging can confirm the clinical suspicion of PeVD by identifying the presence of reflux, insufficiency, compression, or obstruction in the abdomino-pelvic and upper leg/inner thigh veins. For patients with superficial varicose veins as the only symptom, the relation between these (leg) varicose veins and the pelvic floor (extensions from these veins from the upper leg into the pelvis) is an important sign to identify. In the presence of pelvic insufficiency with connections to the leg via the so-called escape points of the pelvic floor, isolated treatment of leg varicosities is likely to result in fast recurrent disease as the underlying pathophysiology (increased pressure from the pelvis) is not resolved: these patients need pelvic intervention. Patients without these insufficient escape points will most likely benefit from isolated treatment of the leg varicosities without pelvic intervention.

## Duplex Ultrasound and Transvaginal Ultrasound (TVUS)

**Fig. 11:**
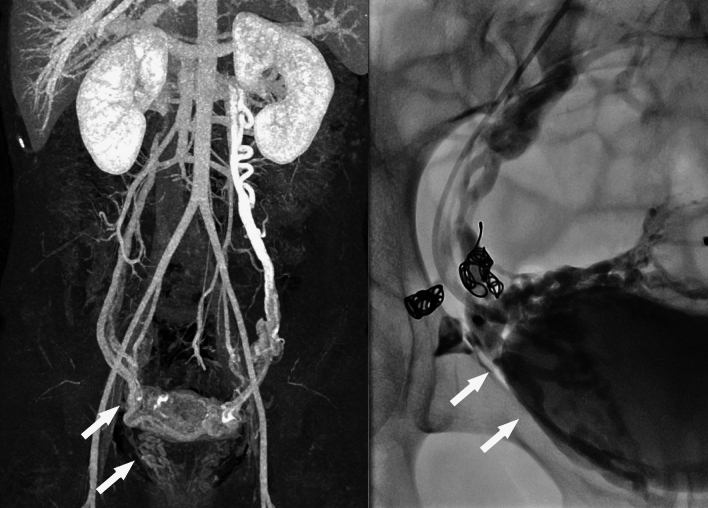
TVUS image of ovarian vein reflux (left) and transabdominal DUS image of parauterine varicosities (right)

DUS is the starting point of the evaluation because of it is in office availability and the potential for dynamic and physiologic evaluations. In these patients, in particular upper leg and labial/vulvar region varicose veins and their relationship with the pelvic floor can be evaluated accurately with DUS. While performing these US techniques are uncommon for interventionalists and/or radiologists, phlebologists and vascular technicians specialized in venous evaluation, routinely use DUS specifically in these areas. It is possible to establish the degree of reflux through the so-called pelvic escape points. Approximately 70% of patients with pelvic veins at the level of the pelvic floor with a diameter of > 5 mm had lower limb varicose veins, in particular in the upper thigh region [37] (Fig. 11).

There are skilled vascular technicians and clinicians capable of virtually assessing the entire venous system of the pelvis and abdomen using transabdominal ultrasound [38]. Alternatively, transvaginal ultrasound can be used to assess the pelvic veins, but neither approach is to be considered the established standard of practice. There have been no prospective studies, but there are many retrospective studies which compare DUS findings to venographic observations. These have been correlated to confirmed diagnoses and/or positive embolization outcomes [38]. Furthermore, there are experience-based results detailing specific predictive value. For transabdominal DUS, a positive predictive value was attributed to ovarian vein caliber. 71.2% at > 5 mm and 83.3% at > 6 mm. Reflux in the ovarian vein was found to have a negative predictive value of 100% if absent (specificity 75%) [39].

In TVUS examinations, the presence of dilated, tortuous para-uterine veins (varicose veins in the pelvis) was 100% sensitive, parametrial veins crossing the uterine body with a diameter > 5 mm 91% [40, 41]. It is important to put these diameter-based results into perspective of the study outcomes listed in the next paragraphs. Diameter assessment might not be as precise or valuable as these, keep in mind retrospective, study outcomes suggest.

## Computed Tomography/Computed Tomography Venography

**Fig. 12:**
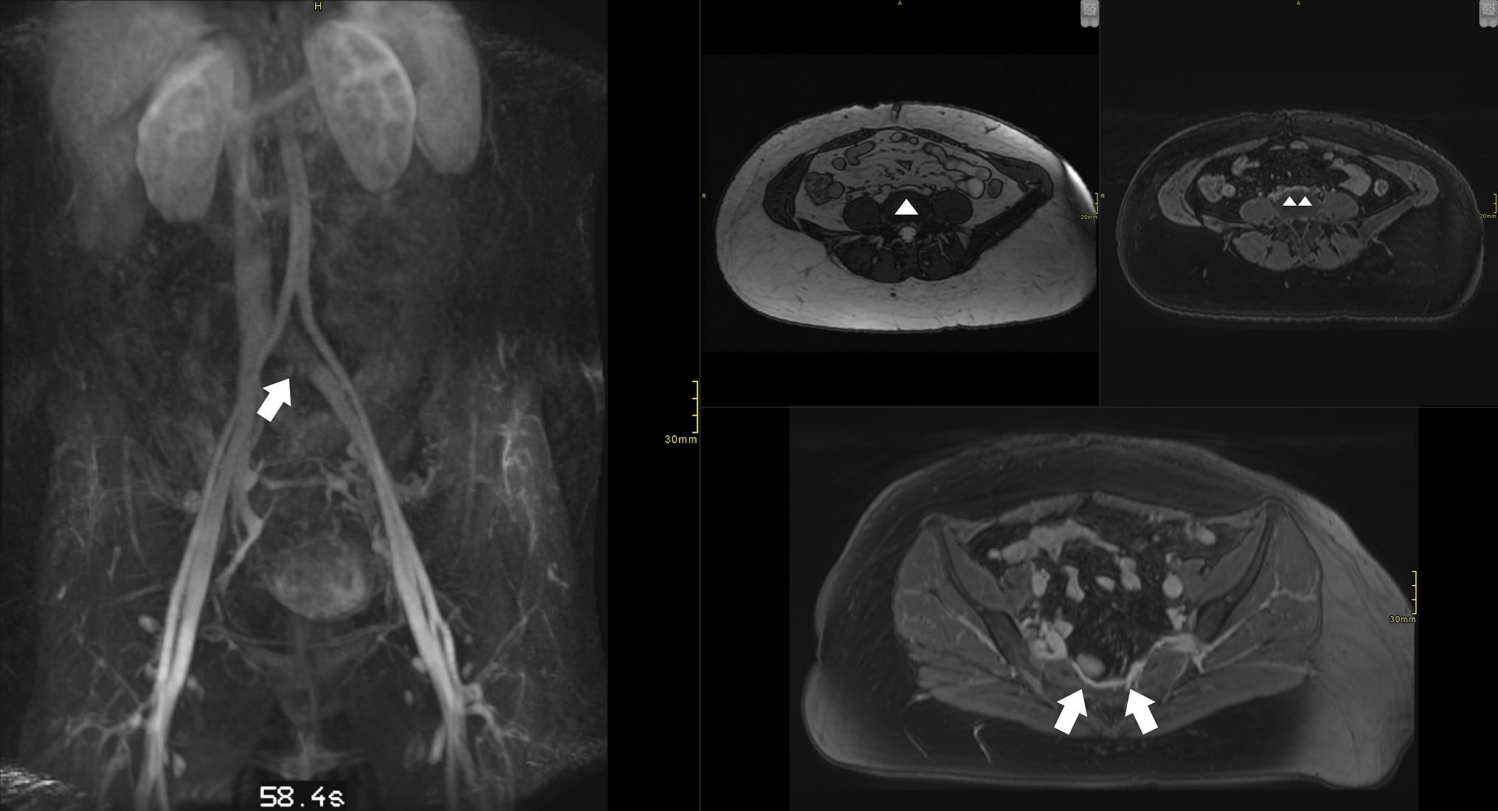
CTV reconstruction of a patient with both ovarian and para-uterine reflux on both sides. Venography confirms the dilated uterine plexus with initial coils placed in side-branches of the uterine plexus and internal pudendal vein plexus

For PeVD, the aim is to identify varicose structures around the uterus, ovaries and in the venous plexuses of the pelvis and pelvic floor. CT scans are frequently used to evaluate the abdomen for pathology so other etiologies of chronic pelvic pain can easily be identified. In addition to a base high special resolution, the addition of intravenous contrast (indirect CTV) allows for a better vessel analysis (Fig. 12).

A limitation of CT (and MRI) is that scanning is limited to the supine (or prone) positioning. Recreation of flow dynamics normally seen in venous reflux (influence of gravity on incompetence in the upright position) is thus hampered. Nevertheless, CT has been used to classify reflux [42]. Comparable to the reported DUS studies, on CTV, dilated left ovarian veins or parametrial veins had more dyspareunia and menstrual symptoms. In particular in patients diagnosed with PevD rather than other defined causes of CPP [43]. However, there are also studies that have shown a high incidence of incidental, asymptomatic venous congestion. One study showed that in asymptomatic women of reproductive age, close to 50% had dilated pelvic and/or ovarian veins ranging from 7 to 12 mm. [44] In addition to the evaluation of varicose veins, the evaluation of (iliac vein) stenosis is important in PeVD. Kuo et al. reported on the relation between left iliac vein cross-sectional area ratios (area of narrowing: area of most peripheral segment) and the degree of pelvic reflux on digital subtraction venography (DSA) providing potentially more non-invasive utility for CTV.

## Magnetic Resonance Imaging/Magnetic Resonance Venography

**Fig. 13:**
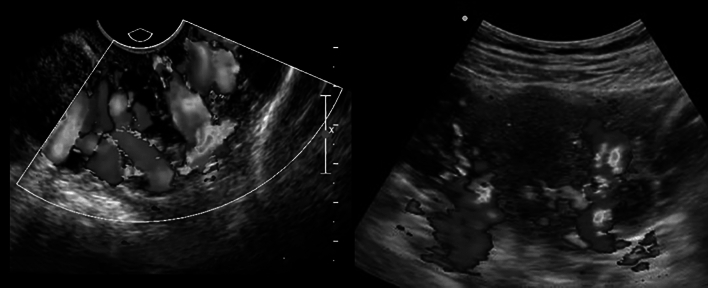
Example of MRV with ovarian (arrows) and para-uterine plexus (arrowheads) insufficiency and correlation with preprocedural findings on venography

**Fig. 14:**
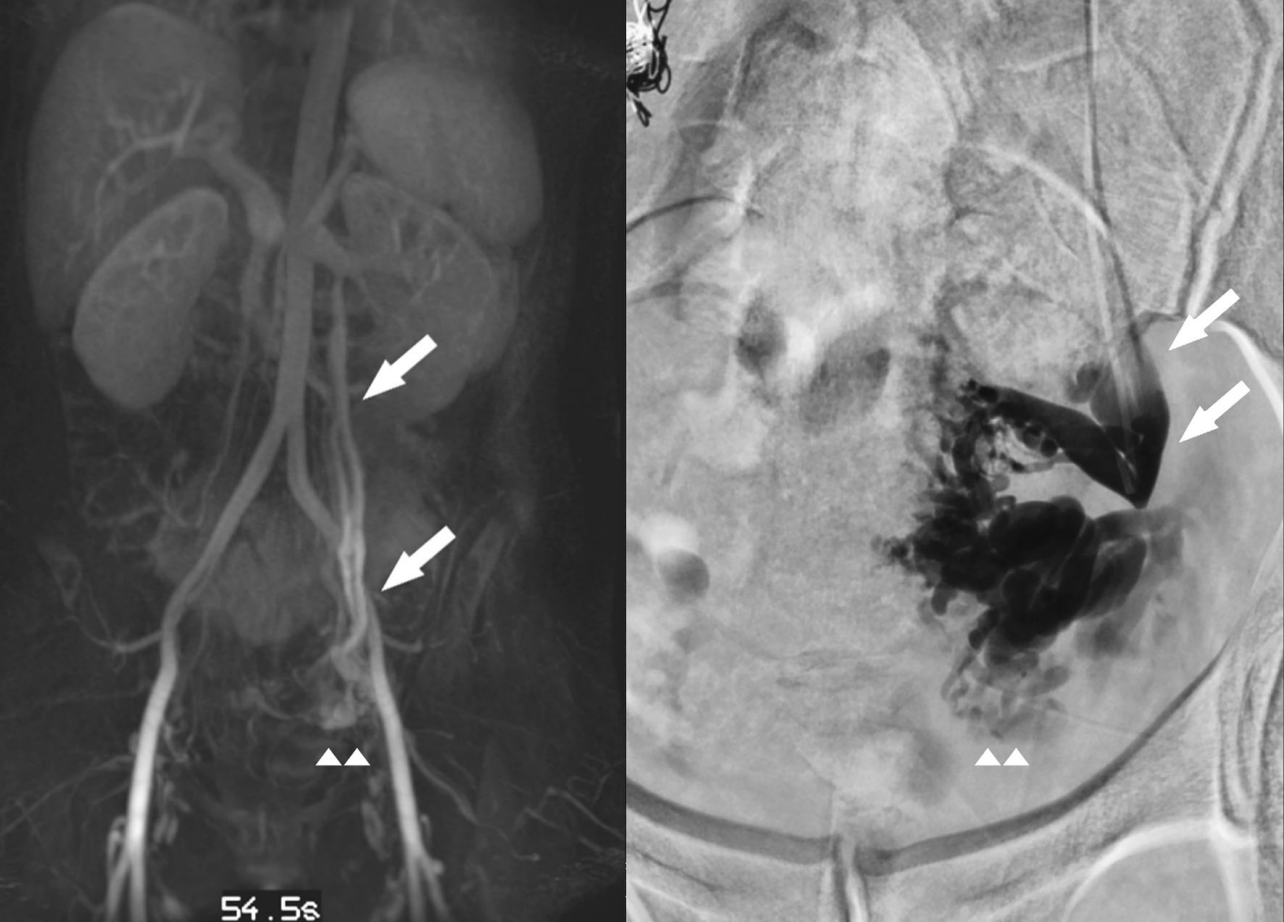
MRV appearance of a common iliac vein stenosis. Left: Dynamic MRV image showing a lack of signal intensity in the left common iliac vein (arrow). Top right: non-contrast True FISP axial image of the stenotic left common iliac vein (arrowhead) and post-contrast VIBE image of the stenotic left common iliac vein (arrowheads). Bottom right: dilated pre-sacral plexus as a confirmation of outflow impairment of the left common iliac vein (arrows)

**Fig. 15:**
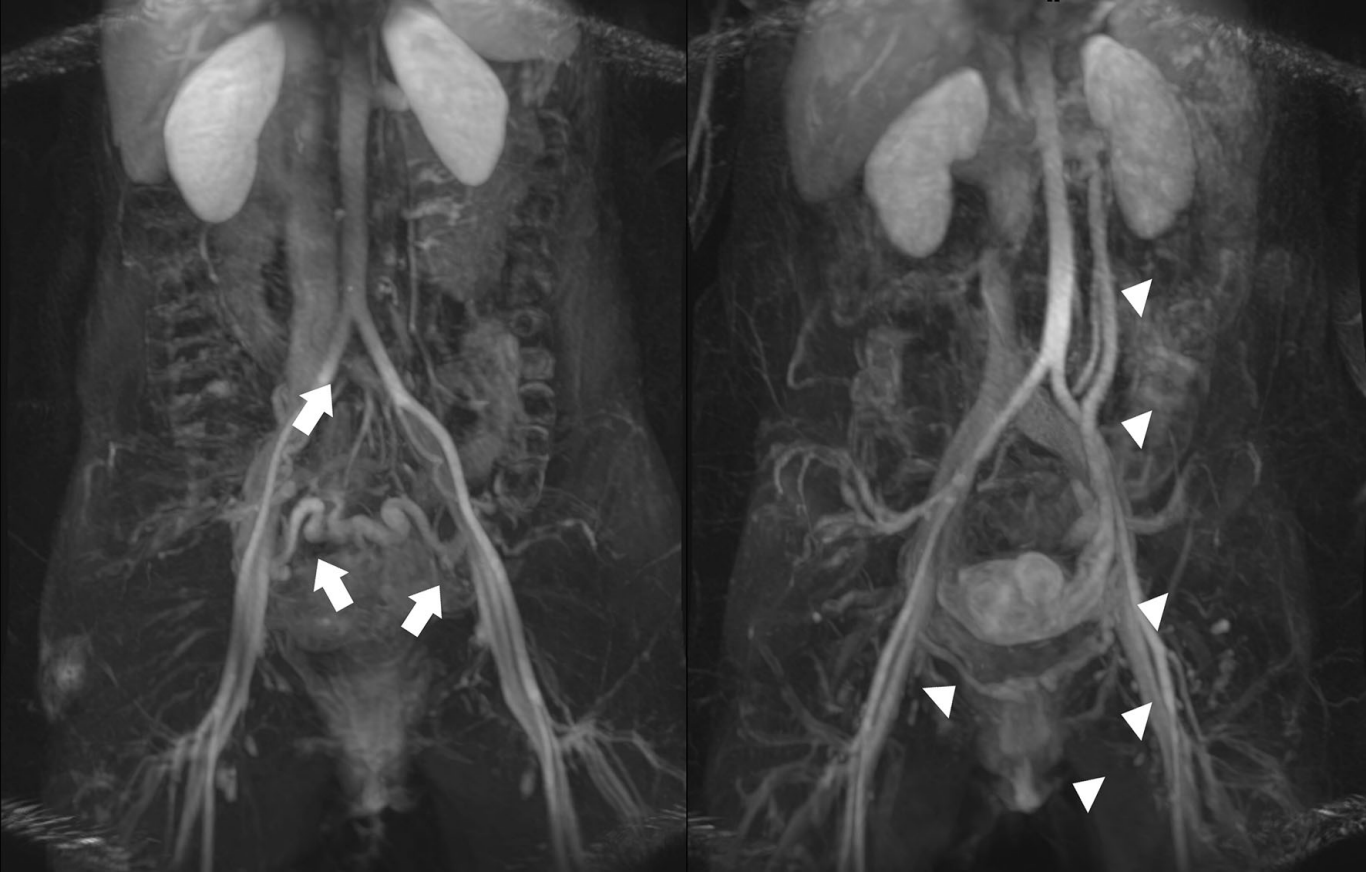
Dynamic MRV evaluation of obstruction (left) versus reflux (right). Left common iliac vein obstruction (arrow) with pudendal and presacral dilation in the pelvis (arrows). Right image shows insufficiency of the left ovarian vein, with para-uterine and internal pudendal veins on both sides affected (arrowheads). There are labial varicosities. There are no signs of an iliac vein stenosis or obstructive pattern

Both with and without contrast, MRI is being utilized to evaluate patients with suspected PeVD. It is a non-ionizing radiation alternative, with reliable anatomic and reflux analysis. For many of the alternative diagnosis to PeVD, it is the optimal modality for assessment. Adding (relative) dynamic evaluation options to the MRV protocol provides it with a clear edge over CT and DUS in the abdominopelvic regions analyzing PeVD [45, 46]. (Figs. 13, 14, 15).

Access to MRI scans is highly variable around the globe. While utilization in centers with access and experience is growing, there is still not enough data demonstrating its value and allowing for a more ‘mainstream’ adoption and solid recommendations in guidelines. Nevertheless, MRI is a favorable alternative in those where US cannot successfully image or evaluate the pelvic venous structures, offering utility to diagnose and map out venous disease prior to an intervention. With MRV, pelvic vessel caliber, reflux, obstruction, and patterns of varicose veins can be evaluated in addition to an anatomical evaluation of the vessels and surrounding (soft) tissues in the pelvis. High sensitivities for ovarian (88%), hypogastric (100%) and pelvic plexus (91%) insufficiency have been reported when compared to conventional venography [46]. Current evidence has shown (dynamic) MRV to be able to grade reflux and caliber estimates correlated well with DUS and venography findings [45–47].

Furthermore, evaluation of (ovarian) vein flow velocities on time resolved MRA/MRV matched venography findings and merits further investigations into the potential for (dynamic) flow measurements. [48]

## Venography and Intravascular Ultrasound (IVUS)

There are generally 2 approaches to identify PeVD. In the first approach, clinicians rely on clinical and DUS information to identify PVI and they do not routinely use CTV or MRV to evaluate these patients. Alternatively, clinicians opt for CTV and MRV as a routine part of their diagnostic work-up. One benefit of routine CTV/MRV examinations is the fact that this aids interventionalists in a better understanding of the anatomy, in particular in cases of anatomical variations which are present in up to 30% of cases. This information can limit fluoroscopy time and complications during catheterization increasing the procedural safety for the patient as well as the treating physician and staff (lower radiation dose). The only role IVUS might have in PeVD is iliac vein stenosis assessment as has been investigated in the VIDIO trial: a > 4-point reduction in the revised Venous Clinical Severity Score between baseline and 6 months as an indicator of clinically meaningful improvement, demonstrated that a cross-sectional area decrease of > 54% by IVUS examination had the highest sensitivity (83% sensitivity, 47% specificity). In contrast, a greater than 52% reduction in diameter by venography had the highest specificity (50% sensitivity, 71% specificity) [29].

While the presented information in this article is based on the available literature and the clinical experience that physicians benefit from the imaging information provided by DUS, TVUS, CTV, MRV, IVUS and venography, it is important to keep in mind that there is a general lack of validated criteria for all the imaging modalities discussed in this article with regard to PeVD.

## Conclusion

Venous disease encompasses a broad spectrum of vascular abnormalities for which, the various imaging modalities described in this article, are more or less suitable for assessment depending on the anatomic level of the abnormalities.

In general, the power of a careful non-invasive analysis by combining information from different modalities about both the superficial and deep venous system lies in the fact that in as few cases as possible patients are exposed to more invasive primarily diagnostic procedures with iodinated contrast and ionizing radiation. Future research should aid us in being able to assess venous disease in a completely non-invasive manner, providing interventionalists with an optimal roadmap for those patients eligible for treatment, resulting in the best outcomes.

## References

[1] Heijboer H, Buller HR, Lensing AWA, et al. A comparison of real-time compression ultrasonography with impedance plethysmography for the diagnosis of deep-vein thrombosis in symptomatic outpatients. N Engl J Med. 1993;329:391365–9. 10.1056/NEJM199311043291901 8413431

[2] Kraaijenhagen RA, Lensing AW, Wallis JW, et al. Diagnostic management of venous thromboembolism. Baillières Clin Haematol. 1998;11:541–86. 10331093 10.1016/s0950-3536(98)80083-8

[3] Garcia-Bolado A, Del Cura JL. CT venography vs ultrasound in the diagnosis of thromboembolic disease in patients with clinical suspicion of pulmonary embolism. Emerg Radiol. 2007;14(6):403–9. 17653779 10.1007/s10140-007-0654-5

[4] Sampson FC, Goodacre SW, Thomas SM, et al. The accuracy of MRI in diagnosis of suspected deep vein thrombosis: systematic review and meta-analysis. Eur Radiol. 2007;17(1):175–81. 16628439 10.1007/s00330-006-0178-5

[5] Barritt DW, Jordan SC, Brist MB. Anticoagulant drugs in the treatment of pulmonary embolism: a controlled trial. Lancet. 1960;275:181309–12. 10.1016/s0140-6736(60)92299-6 13797091

[6] Kearon C, Akl EA, Comerota AJ, et al. Antithrombotic therapy for VTE disease: Antithrombotic Therapy and Prevention of Thrombosis, 9th ed: American College of Chest physicians evidence-based clinical practice guidelines. Chest. 2012;141(2 Suppl):e419S-e496S. 22315268 10.1378/chest.11-2301 PMC3278049

[7] Hull RD, Raskob GE, Brant RF, et al. Relation between the time to achieve the lower limit of the APTT therapeutic range and recurrent venous thromboembolism during heparin treatment for deep vein thrombosis. Arch Intern Med. 1997;157:2562–8. 9531224

[8] Kearon C, Kahn SR, Agnelli G, et al. Antithrombotic therapy for venous thromboembolic disease: American College of Chest physicians evidence-based clinical practice guidelines (8th Edition). Chest. 2008;133:454S-545S. 18574272 10.1378/chest.08-0658

[9] Prandoni P, Lensing AW, Prins MH, et al. Below-knee elastic compression stockings to prevent the post-thrombotic syndrome: a randomized, controlled trial. Ann Intern Med. 2004;141:249–56. 15313740 10.7326/0003-4819-141-4-200408170-00004

[10] Rinfret F, Gu C, Vedantham S, et al. New and known predictors of the postthrombotic syndrome: a subanalysis of the ATTRACT trial. Res Pract Thromb Haemost. 2022;6(6):e12796. 36051541 10.1002/rth2.12796 PMC9424505

[11] Bates SM, Jaeschke R, Stevens SM, et al. Diagnosis of DVT: Antithrombotic Therapy and Prevention of Thrombosis, 9th ed: American College of Chest physicians evidence-based clinical practice guidelines. Chest. 2012;141(2 Suppl):e351S-e418S. 22315267 10.1378/chest.11-2299 PMC3278048

[12] Spiezia L, Campello E, Simion C, et al. Risk factors for post-thrombotic syndrome in patients with a first proximal deep venous thrombosis treated with direct oral anticoagulants. Angiology. 2022;73(7):649–54. 34989625 10.1177/00033197211070889

[13] Magnussen M, Eriksson BI, Kabelo P, et al. Is colour Doppler ultrasound a sensitive screening method in diagnosis deep vein thrombosis after hip surgery? Thromb Haemost. 1996;75(2):242–5. 8815568

[14] Goodacre S, Sampson F, Thomas S, et al. Systematic review and meta-analysis of the diagnostic accuracy of ultrasonography for deep vein thrombosis. BMC Med Imaging. 2005;5:6. 16202135 10.1186/1471-2342-5-6 PMC1262723

[15] Ten Cate-Hoek AJ, van der Velde EF, Toll DB, et al. Common alternative diagnoses in general practice when deep venous thrombosis is excluded. Neth J Med. 2012;70(3):130–5. 22516577

[16] Van Dam LF, Dronkers CEA, Gautam G, Theia Study Group, et al. Magnetic resonance imaging for diagnosis of recurrent ipsilateral deep vein thrombosis. Blood. 2020;135(16):1377–85. 32016390 10.1182/blood.2019004114

[17] Coche EE, Hamoir XL, Hammer FD, et al. Using dual-detector helical CT angiography to detect deep venous thrombosis in patients with suspicion of pulmonary embolism: diagnostic value and additional findings. Am J Roentgenol. 2001;176(4):1035–9. 11264105 10.2214/ajr.176.4.1761035

[18] Loud PA, Katz DS, Bruce DA, et al. Deep venous thrombosis with suspected pulmonary embolism: detection with combined CT venography and pulmonary angiography. Radiology. 2001;219(2):498–502. 11323478 10.1148/radiology.219.2.r01ma26498

[19] Wildberger JE, Mahnken AH, Das M, et al. CT imaging in acute pulmonary embolism: diagnostic strategies. Eur Radiol. 2005;15(5):919–29. 15662491 10.1007/s00330-005-2643-y

[20] Haage P, Krings T, Schmitz-Rode T. Nontraumatic vascular emergencies: imaging and intervention in acute occlusion. Eur Radiol. 2002;12(11):2627–43. 12386751 10.1007/s00330-002-1615-8

[21] Coelho A, O’Sullivan G. Usefulness of direct computed tomography venography in predicting inflow for venous reconstruction in chronic post-thrombotic syndrome. Cardiovasc Intervent Radiol. 2019;42(5):677–84. 30627773 10.1007/s00270-019-02161-5

[22] Kelly J, Hunt BJ, Moody A. Magnetic resonance direct thrombus imaging: a novel technique for imaging venous thromboemboli. Thromb Haemost. 2003;89(5):773–82. 12719772

[23] Laissy J-P, Cinqualbre A, Loshkajian A, et al. Assessment of deep venous thrombosis in the lower limbs and pelvis: MR venography versus duplex Doppler sonography. Am J Roentgenol. 1996;167(4):971–5. 8819396 10.2214/ajr.167.4.8819396

[24] Shankar KR, Abernethy LJ, Das KSV, et al. Magnetic resonance venography in assessing venous patency after multiple venous catheters. J Pediatr Surg. 2002;37(2):175–9. 11819194 10.1053/jpsu.2002.30249

[25] Arnoldussen CWKP, Notten P, Brans R, et al. Clinical impact of assessing thrombus age using magnetic resonance venography prior to catheter-directed thrombolysis. Eur Radiol. 2022;32(7):4555–64. 35347362 10.1007/s00330-022-08599-5 PMC9213279

[26] Carpenter JP, Holland GA, Baum RA, et al. Magnetic resonance venography for the detection of deep venous thrombosis: comparison with contrast venography and duplex Doppler ultrasonography. J Vasc Surg. 1993;18(5):734–41. 8230557 10.1067/mva.1993.49364

[27] Fu Q, Cheng Q, Wu S, et al. Fat-suppressed magnetic resonance volume interpolated examination for deep venous thrombosis compared with duplex sonography. Exp Ther Med. 2020;19(4):2632–40. 32256744 10.3892/etm.2020.8500 PMC7086293

[28] Gagne PJ, Tahara RW, Fastabend CP, Dzieciuchowicz L, Marston W, Vedantham S, Ting W, Lafrati MD. Venography versus intravascular ultrasound for diagnosing and treating iliofemoral vein obstruction. J Vasc Surg Venous Lymphat Disord. 2017;5(5):678–87. 28818221 10.1016/j.jvsv.2017.04.007

[29] Gagne PJ, Gasparis A, Black S, Thorpe P, Passman M, Vedantham S, Marston W, Lafrati LD. Analysis of threshold stenosis by multiplanar venogram and intravascular ultrasound examination for predicting clinical improvement after iliofemoral vein stenting in the VIDIO trial. J Vasc Surg Venous Lymphat Disord. 2018;6(1):48–56. 29033314 10.1016/j.jvsv.2017.07.009

[30] Saleem T, Raju S. Comparison of intravascular ultrasound and multidimensional contrast imaging modalities for characterization of chronic occlusive iliofemoral venous disease: a systemtic review. J Vasc Surg Venous Lymphat Disord. 2021;9(6):1545-1556.e2.33965613 10.1016/j.jvsv.2021.03.022

[31] Khairy SA, Neves RJ, Hartung O, O’Sullivan GJ. Factors associated with contralateral deep venous thrombosis after iliocaval venous stenting. Eur J Vasc Endovasc Surg. 2017;54(6):745–51. 10.1016/j.ejvs.2017.07.011. 28886989 10.1016/j.ejvs.2017.07.011

[32] Meissner MH, Khilnani NM, Labropoulos N, et al. The Symptoms-varices-pathophysiology classification of pelvic venous disorders: a report of the American Vein & Lymphatic Society International Working Group on pelvic venous disorders. J Vasc Surg Venous Lymphat Disord. 2021;9(3):568–84. 33529720 10.1016/j.jvsv.2020.12.084

[33] Balabuszek K, Torobek M, Picture R. Comprehensive overview of the venous disorder known as pelvic congestion syndrome. Ann Med. 2022;54(1):22–36. 34935563 10.1080/07853890.2021.2014556 PMC8725876

[34] Jurga-Karwacka A, Karwacki GM, Schoetzau A, et al. A forgotten disease: pelvic congestion syndrome as a cause of chronic lower abdominal pain. PLoS ONE. 2019;14(4):e0213834. 30939134 10.1371/journal.pone.0213834 PMC6445463

[35] Daniels JP, Champaneria R, Shah L, Gupta JK, Birch J, Moss JG. Effectiveness of embolization or sclerotherapy of pelvic veins for reducing chronic pelvic pain: a systematic review. J Vasc Interv Radiol. 2016;27(10):1478-1486.e8. 27397619 10.1016/j.jvir.2016.04.016

[36] Phillips D, Deipolyi AR, Hesketh RL, et al. Pelvic congestion syndrome: etiology of pain, diagnosis, and clinical management. J Vasc Interv Radiol. 2014;25(5):725–33. 24745902 10.1016/j.jvir.2014.01.030

[37] Balian E, Lasry JL, Coppé G. Pelviperineal venous insufficiency and varicose veins of the lower limbs. Phlebolymphology. 2008;5:17–26.

[38] Labropoulos N, Borge M, Pierce K, Pappas PJ. Criteria for defining significant central vein stenosis with duplex ultrasound. J Vasc Surg. 2007;46:101–7. 17540535 10.1016/j.jvs.2007.02.062

[39] Steenbeek MP, van der Vleuten CJM, Schultze Kool LJ, Nieboer TE. Noninvasive diagnostic tools for pelvic congestion syndrome: a systematic review. Acta Obstet Gynecol Scand. 2018;97(07):776–86. 29381188 10.1111/aogs.13311 PMC6033028

[40] Park SJ, Lim JW, Ko YT, Lee DH, Yoon Y, Oh JH. Diagnosis of pelvic congestion syndrome using transabdominal and transvaginal sonography. AJR Am J Roentgenol. 2004;182:683–8. 14975970 10.2214/ajr.182.3.1820683

[41] Giacchetto C, Cotroneo GB, Marincolo F, Cammisuli F, Caruso G, Catizone F. Ovarian varicocele: ultrasonic and phlebographic evaluation. J Clin Ultrasound. 1990;18(07):551–5. 2170453 10.1002/jcu.1870180705

[42] Iromura T, Nishioka T, Nishioka S, Ikeda H, Tomita K. Reflux in the left ovarian vein: analysis of MDCT findings in asymptomatic women. AJR Am J Roentgenol. 2004;183(05):1411–5. 15505313 10.2214/ajr.183.5.1831411

[43] Awad AS, Taha MMM, Manaf MHA. Role of multi-detector CT venography in evaluation of pelvic congestion syndrome. Egypt J Radiol Nucl Med. 2020;51:159.

[44] Rozenblit AM, Ricci ZJ, Tuvia J, Amis ES. Jr Incompetent and dilated ovarian veins: a common CT finding in asymptomatic parous women. AJR Am J Roentgenol. 2001;176(01):119–22. 11133549 10.2214/ajr.176.1.1760119

[45] Dick EA, Burnett C, Anstee A, Hamady M, Black D, Gedroyc WMW. Time-resolved imaging of contrast kinetics three-dimensional (3D) magnetic resonance venography in patients with pelvic congestion syndrome. Br J Radiol. 2010;83(994):882–7. 20846985 10.1259/bjr/82417499 PMC3473747

[46] Yang DM, Kim HC, Nam DH, Jahng GH, Huh CY, Lim CW. Time-resolved MR angiography for detecting and grading ovarian venous reflux: comparison with conventional venography. Br J Radiol. 2012;85(1014):e117–22. 21385913 10.1259/bjr/79155839 PMC3474099

[47] Asciutto G, Mumme A, Marpe B, Köster O, Asciutto KC, Geier B. MR venography in the detection of pelvic venous congestion. Eur J Vasc Endovasc Surg. 2008;36(04):491–6. 18718774 10.1016/j.ejvs.2008.06.024

[48] Meneses LQ, Uribe S, Tejos C, Andía ME, Fava M, Irarrazaval P. Using magnetic resonance phase-contrast velocity mapping for diagnosing pelvic congestion syndrome. Phlebology. 2011;26(04):157–61. 21690172 10.1258/phleb.2010.010049

